# Berberine in Human Oncogenic Herpesvirus Infections and Their Linked Cancers

**DOI:** 10.3390/v13061014

**Published:** 2021-05-28

**Authors:** Miroslava Šudomová, Kateřina Berchová-Bímová, Stefania Marzocco, Alena Liskova, Peter Kubatka, Sherif T.S. Hassan

**Affiliations:** 1 Museum of Literature in Moravia, Klášter 1, 66461 Rajhrad, Czech Republic; sudomova@post.cz; 2 Department of Applied Ecology, Faculty of Environmental Sciences, Czech University of Life Sciences Prague, Kamýcká 129, 16500 Prague, Czech Republic; berchova@fzp.czu.cz; 3 Department of Pharmacy, University of Salerno, 84084 Fisciano, SA, Italy; smarzocco@unisa.it; 4 Department of Obstetrics and Gynecology, Jessenius Faculty of Medicine, Comenius University in Bratislava, 03601 Martin, Slovakia; alenka.liskova@gmail.com; 5 Department of Medical Biology, Jessenius Faculty of Medicine, Comenius University in Bratislava, 03601 Martin, Slovakia; kubatkap@gmail.com

**Keywords:** berberine, oncogenic herpesviruses, cancer, Kaposi’s sarcoma-associated herpesvirus, Epstein–Barr virus, herpes simplex virus, human cytomegalovirus, inflammation

## Abstract

Human herpesviruses are known to induce a broad spectrum of diseases, ranging from common cold sores to cancer, and infections with some types of these viruses, known as human oncogenic herpesviruses (HOHVs), can cause cancer. Challenges with viral latency, recurrent infections, and drug resistance have generated the need for finding new drugs with the ability to overcome these barriers. Berberine (BBR), a naturally occurring alkaloid, is known for its multiple biological activities, including antiviral and anticancer effects. This paper comprehensively compiles all studies that have featured anti-HOHV properties of BBR along with promising preventive effects against the associated cancers. The mechanisms and pathways induced by BBR via targeting the herpesvirus life cycle and the pathogenesis of the linked malignancies are reviewed. Approaches to enhance the therapeutic efficacy of BBR and its use in clinical practice as an anti-herpesvirus drug are also discussed.

## 1. Introduction

Herpesviruses are a diverse group of large double-stranded DNA viruses that share a common virion morphology. Herpesviruses, which belong to the *Herpesviridae* family, are highly infectious and frequently infect humans for life and persist latently along with the ability to cause recurrent infections [1,2]. Some types of herpesvirus that can lead to cancer are recognized to be oncogenic, such as Epstein–Barr virus (EBV, also known as human herpesvirus 4) and Kaposi’s sarcoma-associated herpesvirus (KSHV, also known as human herpesvirus 8) [3,4]. On the other hand, some studies have reported other potentially human oncogenic herpesviruses (HOHVs) with an effect on various types of cancer such as herpes simplex virus 1 (HSV-1, also known as human herpesvirus 1), herpes simplex virus 2 (HSV-2, also known as human herpesvirus 2), and human cytomegalovirus (HCMV) [5,6,7]. It is known that herpesviruses use the infection strategy ‘‘run and hide,’’ and significant complications of these infections can be noticed once the immune system is compromised by various factors that negatively affect the immune system, including physiological and environmental factors [8,9]. Infections with herpesviruses are currently challenging to cure, and the clinical drugs used to treat them, such as acyclovir and other nucleoside analogs, do not entirely cure the disease or prevent recurrent infections while blocking the viral replication, thus reducing the duration of symptoms and promoting the healing of epithelial damage, lesions, and other cellular damages that were triggered by virus infection [10,11]. On the other hand, the overuse of these drugs has generated the problem of drug resistance, which in turn has adversely affected the treatment efficacy [12]. Currently, anti-herpesvirus drug development strategies face many challenges, and the most important tasks are linked with developing potent anti-herpesvirus medicines that can conquer the problems of drug resistance, viral latency, and recurrent infections and can also act with diverse mechanisms of action, minimum or no toxicity, and minimum adverse effects [13,14]. Most drug discovery strategies rely on natural products as a considerable source of new drug candidates with relatively safe profiles, especially from plant sources [15].

**Figure 1:**
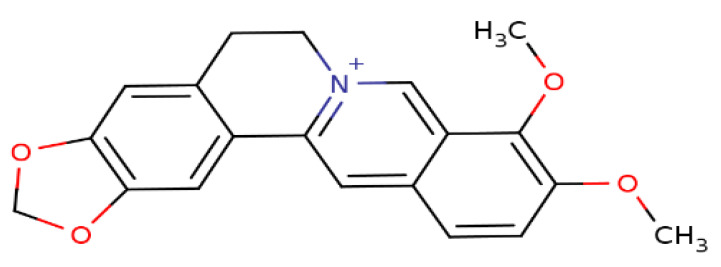
Chemical structure of berberine.

Berberine (BBR), a secondary metabolite that is biosynthesized by various plant species and is commonly present in the roots, rhizomes, and stem barks of various Chinese herbs as well as several plants of the *Berberis* genus [16]. Chemically, this compound is a quaternary ammonium salt of an isoquinoline alkaloid (PubChem CID: 2353) with a molecular weight of 336.4 g/mol (Figure 1) [17]. Biologically, BBR in numerous preclinical and limited human studies has been proven to exert various beneficial bioactivities against several human diseases, including microbial infections, inflammation, various types of cancer, cardiovascular diseases, gastrointestinal disorders, neurodegenerative diseases, depression, and metabolic dysfunctions [16,18,19,20]. More information about berberine’s bioavailability and safety profile is discussed in a later section.

Considering the medicinal effects of BBR on several infectious diseases, in this review we aim to comprehensively document all findings that reveal the anti-herpesvirus properties and potential therapeutic value of this biomolecule against HOHV infections and their linked cancers. The mechanisms and pathways by which BBR causes anti-herpesvirus actions by targeting multiple stages during the herpesvirus life cycle and the mechanisms employed via targeting the pathogenesis of the associated cancers are documented. Also, in this review, we discuss various strategies to improve the therapeutic properties of BBR as well as various options that could enhance its use in clinical practice. Online databases such as PubMed, Scopus, Web of Science Core Collection, SciFinder, ScienceDirect, and Google Scholar were employed to perform the literature search, utilizing terms that characterize berberine, human herpesvirus infections, and their associated cancers. This review covers investigations published in the years from 2007 to 2021.

## 2. General Overview of Herpesviruses and Their Infection Strategies

Human herpesviruses are highly infectious agents that cause critical health complications to humans, and herpesvirus host cells are characterized by their susceptibility to productive or latent infections [21]. The host’s immune system plays an essential role in the fight against diseases, including viral infections. Once the primary infection originates, the productive infection process takes place, which is then restricted by the host immune response, leaving behind latently infected cells that endure in the host [22,23]. During latency, several viral genes are expressed, and the viral genomes usually persist as episomes in the nuclei of infected cells. However, viral genomes also have the ability to incorporate into the host genome [24]. It is known that herpesviruses infect different cell types in various tissues, and according to the type of cell and tissue tropism, these viruses are sub-classified [25]. For instance, alpha-herpesviruses (such as HSV) induce latency in the cells of the nervous system, while beta-herpesviruses (such as HCMV) are described with broad cell tropism and can develop latency in progenitors of the hematopoietic cell system. Gamma-herpesviruses (such as EBV and KSHV) expose a more restricted cell tropism and can become latent in the cells with the ability to cause tumors in their infected hosts [24,26,27,28].

The entry site for a particular herpesvirus is usually different from the latency site [29,30]. Accordingly, some tactics are employed by the herpesvirus to reach the site of latency such as using migrating cells as vehicles for dissemination or using cell protrusions of nerve cells (in the case of alpha-herpesviruses). On the other hand, the herpesvirus utilizes the same routes back during the reactivation process to guarantee the horizontal spread from productively infected cells [24,31,32,33,34].

Considering the capability of herpesviruses to control their host cells by altering their differentiation status, understanding the host–virus interactions and all factors that are involved in these interactions is very crucial in herpesvirus research. Such information might help deliver more visions into disease pathogenesis and develop new anti-herpesvirus drugs and vaccines [35,36]. Consequently, extensive efforts have been made to understand more about the interaction of HOHVs and host factors using various computational, analytical, and bioanalytical-based approaches [37,38,39,40,41,42,43,44]. 

## 3. Berberine Targets Clinically Recognized Oncogenic Herpesviruses

Herpesviruses are known to employ several immune evasion strategies to cause latent infections in their host cells with the ability to generate certain types of cancer [45]. The potential of developing cancer has been clinically confirmed with EBV and KSHV infections, which are classified as a class I carcinogen [46]. The major carcinogenic mechanisms employed by EBV and KSHV have recently been clarified, where both viruses can repress apoptosis and tumor suppressor pathways, enhance the oncogenic microenvironment, promote cellular migration, metastasis, and angiogenesis, and generate mutagenesis [3,45,46].

This section records all studies concerning BBR and its protective effects on EBV and KSHV and their associated cancers, with a focus on the mechanisms of action and pathways along with effective concentrations or doses.

### 3.1. Berberine Targets Epstein–Barr Virus and Its Associated Cancers

EBV is a double-stranded DNA virus that belongs to the *gamma-Herpesviridae* subfamily [47] and was first identified in Burkitt’s lymphoma by Sir Anthony Epstein and colleagues in 1964 [48,49]. This pathogen was the first tumor virus discovered in humans and is principally linked with lymphomas and epithelial cell cancers [50]. Saliva exchange is the most known transmitting method for EBV infection and therefore symptomatic initial infection or infectious mononucleosis was described as ‘’kissing disease’’ [51]. There is a strong connection between EBV and the development of cancer, where experimental data confirmed that EBV infection is linked with various human proliferative diseases involving primarily epithelial or lymphoid cells, including nasopharyngeal carcinoma (NPC) [52,53]. EBV demonstrates a type II latency mechanism in NPC patients, and this latency is mainly characterized by the expression of Epstein–Barr nuclear antigen 1 (EBNA1), which was observed to be vital for the replication, partition, transcription, and protection of the viral genome [54,55,56]. Other critical latent membrane proteins and several non-coding RNAs are also expressed by the virus during the latency phase associated with NPC [57]. Therefore, such targets are very crucial in the design of new drug candidates useful in the management of EBV and its linked NPC [58]. 

The direct suppression effect of BBR on EBV infection has been revealed in limited experiments, while its potent antitumor impacts have been explored in numerous in vitro and in vivo studies evaluated in multiple EBV positive-cancerous cells. For instance, in preclinical investigations (in vitro and in vivo), Wang and coauthors [59] showed the capacity of BBR to inhibit the latent and lytic replication of EBV-positive NPC cells and reduce cell proliferation, cause cell cycle arrest, and promote apoptosis in the EBV-positive NPC cells (Table 1). Their results unveiled diverse mechanisms of action at multiple molecular and cellular levels, suggesting BBR as a promising drug for the therapies of EBV infection and EBV-associated tumors such as NPC. In an in vivo experiment using athymic nude mice, BBR was observed to efficiently hinder the tumorigenicity and growth of EBV-positive NPC cells. The mechanism has been found to correlate with successful inhibition of signal transducer and activator of transcription 3 (STAT3) activation in NPC cells [60]. On the other hand, this study did not determine the inhibitory action of BBR against EBV. NPC could also be generated in the absence of EBV infection [61]. Since various drugs could work in a synergistic manner and provide enhanced treatment efficacy [62], BBR in combination with ginsenoside Rg3 (Rg3; an active molecule from *Panax ginseng*) was evaluated for improved anticancer properties and was detected to induce remarkable inhibition of NPC cell proliferation (in the absence of EBV infection) in vitro and in vivo. The underlying mechanism has been revealed to affect the mitogen-activated protein kinase (MAPK)/extracellular signal-regulated kinase (ERK) signaling pathways [63]. In another research performed on EBV-transformed B cells and cancerous B cells, treatment with BBR led to significant induction of mitochondrial apoptosis, clarifying that the role of X-linked inhibitor of apoptosis protein-associated factor 1 (XAF1) as a supporter of the mitochondrial apoptosis pathway might offer a novel target for cancer therapy, mainly for cancers with wild-type p53 expression [64].

### 3.2. Berberine Targets Kaposi’s Sarcoma-Associated Herpesvirus and Its Associated Cancers

KSHV was first detected in Kaposi sarcoma (KS) and is associated with around 1% of all human malignancies. KSHV, a gamma-herpesvirus, is transmitted through sex and appears to be spread in other ways, such as through blood and saliva [65,66]. This virus is also linked with other malignancies such as primary effusion lymphoma (PEL) and a subset of multicentric Castleman’s disease (MCD). KS has been the focus of investigation in KSHV research, as it is the most common acquired immunodeficiency syndrome (AIDS)-related malignancy [67,68]. KS is a cancer that originates in lymph or blood vessels and commonly appears as lesions on the skin, the inside of the mouth, or internally. Genetic alterations driven by chromosomal instability represent the main feature of KSHV-associated cancers [69,70]. It has been proved that KSHV can infect several different cell types, including endothelial cells, B cells, epithelial cells, dendritic cells, monocytes, and fibroblasts, and the virus uses a specific strategy to attack the host cell, generating latent and lytic infection [71]. The viral life cycle of KSHV, and the mechanisms of inducing latency, have comprehensively been reviewed by Cesarman and colleagues [72], and we highly recommend the readers to refer to this work for more detailed information. BBR has been involved in extremely limited studies related to KSHV-associated malignancies research, and no antiviral experiments have been performed against KSHV so far.

PEL, a blood cancer, is a large B-cell lymphoma located in the body cavities, described by pleural, peritoneal, and pericardial fluid lymphomatous effusions [73]. In a combined in vitro and in vivo study, Goto et al. [74] examined in depth the antitumor activity of BBR against PEL. Their work employed multiple in vitro biochemical assays, where BBR has successfully inhibited the proliferation of KSHV sequence-positive PEL (BC-1, BCBL-1, and TY-1) cells with 50% inhibitory concentration (IC_50_) values of 13.56, 29.17, and 32.82 µM, respectively. BBR also caused caspase-dependent apoptosis at concentrations of 30 and 100 µM. The mechanisms were found to relate to the ability of BBR to block nuclear factor κB (NF-κB) activation by impeding IκB kinase (IKK) phosphorylation, IκB phosphorylation, and IκB degradation, upstream targets of the NF-κB pathway, in PEL cells. Additionally, the authors used a xenograft mouse model to confirm the in vitro results, where treatment with BBR (at a dose of 10 mg/kg) significantly hindered the growth and invasion of PEL cells compared to untreated mice.

Although we reviewed the promising role of BBR in preventing recurrent EBV infection and its connected cancers and KSHV-associated malignancies as well, additional in-depth studies that incorporate BBR into EBV and KSHV research are needed. Such studies should consider some facts that both viruses can control the host epigenetic machinery and contribute to the early phases of tumor development by initiating oncogenic changes within the cell, with the ability to hide using a strategy “hit and run’’ [75]. These facts make treatment of EBV and KSHV infections hard to efficiently manage. Therefore, we discuss in a later section various approaches to overwhelm these barriers. 

### 3.3. Berberine’s Mechanisms against γ-Herpesviruses and Their Linked Malignancies 

**Figure 2:**
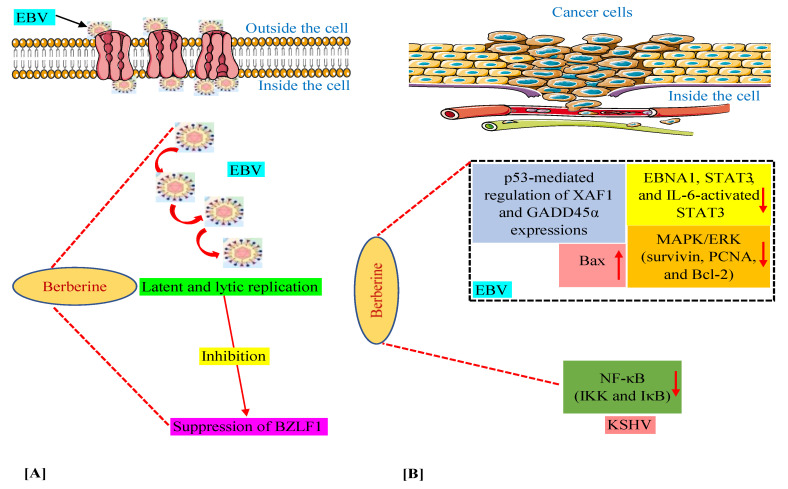
Mechanisms of berberine against EBV replication (**A**) and EBV- and KSHV-associated cancers (**B**). The upward-pointing arrow indicates enhancement/upregulation, and the downward-pointing arrow indicates inhibition/downregulation. EBNA1, Epstein–Barr nuclear antigen 1; EBV, Epstein–Barr virus; GADD45α, growth arrest and DNA damage inducible alpha; IKK, IκB kinase; IL-6, interleukin-6; KSHV, Kaposi’s sarcoma-associated herpesvirus; MAPK/ERK, mitogen-activated protein kinase/extracellular signal-regulated kinase; NF-κB, nuclear factor kappa B; PCNA, proliferating cell nuclear antigen; STAT3, signal transducer and activator of transcription 3; XAF1, X-linked inhibitor of apoptosis protein-associated factor 1.

We have discussed above that in various in vitro and animal experiments, BBR has been shown to modulate the expression of a variety of genes and proteins and target several pathways involved in tumorigenesis connected with EBV and KSHV infections [59,60,63,64,74]. Moreover, we documented the capability of BBR to suppress EBV latent and lytic infection by affecting the expression of the transcription factor BZLF1, which is a critical factor required for viral replication [59]. Thus, in this section, we summarize these mechanisms and pathways in Figure 2, especially for readers who prefer to look at display items. 

## 4. Berberine Targets Other Potentially Oncogenic Herpesviruses

The potential role of alpha-herpesviruses (HSV-1 and HSV-2) and beta-herpesviruses (HCMV) in various types of cancer has been reported in numerous preclinical and clinical trials. For example, infection with HSV-1 has been observed in patients diagnosed with head and neck squamous cell carcinoma (HNSCC); however, the HSV-1 infection was noticed to be asymptomatic [76,77]. In comparative studies, higher HSV shedding was detected in cancer patients who received chemotherapy and had weak immune systems [78,79]. The presence of HSV-1 and HSV-2 infections in patients with HNSCC was evaluated in a clinical study. The results showed that the reactivation of both viruses was observed to be relatively infrequent, indicating that both viruses may have a potential role in the development of HNSCC [80]. Other clinical trials have also evidenced the association between HSV (HSV-1 and HSV-2) infections and other types of cancer as pathogenic cofactors or etiological agents such as cervical, prostate, skin, and oral cancers [81,82,83,84,85]. The oncogenic mechanisms of HSV-1 and HSV-2 have been attributed to their capacity to modulate DNA synthesis and induce mutations. Moreover, both viruses in lytically infected cells expose anti-apoptotic activities, specifically ICP10PK protein, which hinders apoptosis through the activation of the Ras/Raf-1/MEK/ERK pathways [5,6].

HCMV is a non-oncogenic herpesvirus, but various clinical and experimental discoveries have advocated the partial involvement of this virus in the etiology of different malignancies, including NPC, HNSCC, breast cancer, gastric cancer, glioblastoma, and colorectal cancer [86,87,88,89,90,91,92,93]. Several mechanisms have been claimed for the oncogenic effect of HCMV via generating cell cycle progression, chromosomal deviations, and vascular endothelial growth factor (VEGF) expression, activating cell motility and migration, and suppressing DNA damage repair and apoptotic pathways [7,94]. Besides, a new mechanism called oncomodulation has recently been proposed in which the virus infects tumor cells and modulates their malignant activities without affecting cell transformation [93]. The term *oncomodulation* describes the effects of specific viruses on tumor biology to promote cancer aggressiveness and resistance to conventional treatments [7,95]. The oncomodulatory effects of HCMV can be detailed by an example of human colorectal carcinoma. Approximately 40% of colorectal carcinoma specimens were found to be positive for HCMV that correlated with unfavorable outcomes, especially in the elderly. Therefore, HCMV can affect the tumor microenvironment of colorectal carcinoma by altering certain cellular functions and signaling pathways regulating processes essential for carcinogenesis and anticancer immunity. Such oncomodulatory effects of HCMV regulating malignant behavior of colorectal cancer cells are brought about through the modulation of various signaling pathways, growth, and consequent promotion of cell survival, proliferation, and angiogenesis. However, the oncomodulatory activity of HCMV needs to be further investigated systemically [96].

In conclusion, inhibiting the infectivity of these herpesviruses might help prevent the above-mentioned human cancers. Therefore, in this section, we document the contribution of BBR to alpha- and beta-herpesvirus research as an antiherpetic and chemopreventive agent. Also, we point out the mechanisms of action combined with the efficient concentrations or doses.

### 4.1. Berberine Targets Alpha-Herpesviruses

HSV, a human alpha-herpesvirus, is one of the most common viral infections in humans. This virus is categorized into two types, HSV-1 and HSV-2 [97]. Both types can infect orofacial areas and the genital tract, where HSV-1 commonly induces oral herpes and HSV-2 is mostly accountable for genital herpes. HSV-1 is also recognized to cause genital herpes. In most cases, both infections are asymptomatic [98,99].

Several experimental findings have provided diverse insights into the blocking of HSV infections by BBR. In a comprehensive study, inhibition of HSV-1 and HSV-2 replications by BBR was determined using the viral plaque assay combined with multiple biochemical analyses. BBR effectively inhibited both viruses and the inhibitory activity was explained by mechanisms of action mediated by modulating the NF-κB and c-Jun N-terminal kinase (JNK) pathways [100]. Moreover, Chin et al. [101] showed the anti-HSV properties of BBR and its effects on virus adsorption and penetration. Their results concluded that BBR can repress the viral replication cycle by inhibiting late gene products after virus penetration and no later than the viral DNA synthesis stage. Another research group stressed, in an in vitro experiment, that BBR showed slight anti-HSV-2 properties in human vaginal epithelial cells [102]. In addition to isolated BBR, Cortex phellodendri (dried bark of *Phellodendron amurense* Ruprecht) aqueous extract (CP), which contains BBR as the main compound, showed significant immunomodulatory and antiviral activity. CP exhibited an ability to inhibit viral replication, as demonstrated by reduced green fluorescent protein (GFP) expression and viral titers against HSV-GFP upon CP pre-treatment when compared with untreated groups. BBR as a principal constituent of CP exhibited promising antiviral properties similar to CP with a suggested mechanism that involves type I interferon (type I IFN) stimulation [103]. Other preclinical studies have claimed the ability of 13-hexyl-berberine hydrochloride (HB-13), a BBR derivative, to suppress the infectivity of HSV-1 and HSV-2 and treat the herpes lesions [104,105]. Table 2 provides a detailed overview of the above-discussed effects of BBR and its derivatives along with the induced mechanisms against human alpha-herpesviruses. 

### 4.2. Berberine Targets Beta-Herpesviruses (Human Cytomegalovirus)

HCMV is a beta-herpesvirus with highly infectious properties that threatens human health, particularly immunocompromised people. The antiviral properties of BBR have been processed against various HCMV strains.

Recently, in a mechanistic study, Luganini and co-workers [106] have clarified the competency of BBR to strongly suppress the replication of various HCMV strains with a mechanism that targets viral immediate early-2 (IE2) protein transactivating activity. IE2 is a striking target for anti-HCMV drugs as it plays a significant role in the progression of viral replication and reactivation from latency [107]. Moreover, the orally available form of BBR (BBR chloride) exhibited anti-HCMV activity with a proposed mechanism of action that targets some stages in the viral life cycle; however, the detailed mechanism should be clarified by further investigation [108]. Table 3 highlights a detailed overview of the above-discussed capacity of BBR and its mechanisms against HCMV.

## 5. Berberine between Inflammation and Cancer

Herpesvirus infections are often tied with inflammation. This has been observed during the replication process of EBV and HCMV, where both viruses can trigger pro-inflammatory cytokine production and consequently could influence systemic inflammation [109,110]. Similarly, inflammation associated with post-infection complications of oral and genital herpes was reported in diverse medical observations [111,112,113]. Besides, KSHV inflammatory cytokine syndrome (KICS) caused by KSHV has recently been described [114,115].

Inflammation is generated in complex pathways by microbial infections [116]. Once the body recognizes a pathogen attack, the innate immune system acts by producing pro-inflammatory cytokines as a part of a defense system to stop the pathogen and limit the replication process (in the case of viral infection). However, pro-inflammatory cytokines have a negative impact as they play a critical role in inflammatory diseases of infectious origin [117,118]. Modern research has disclosed that nearly 20% of human cancers are related to chronic inflammation caused by infections, where such inflammation can induce multiple DNA and cellular damages and lead to cancer [119,120].

**Figure 3:**
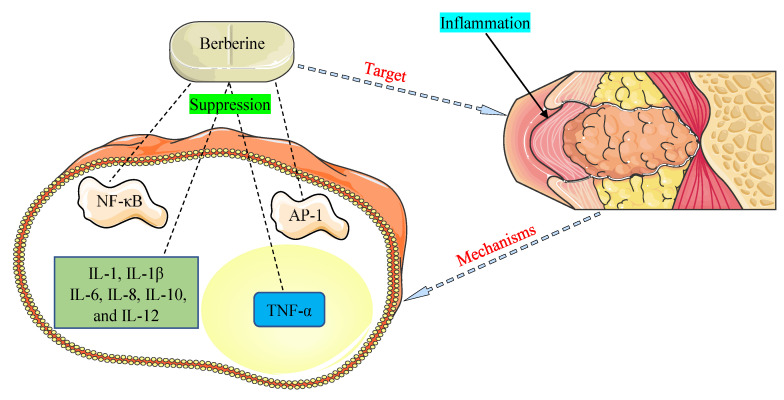
Mechanisms by which berberine demonstrates anti-inflammatory properties against inflammation associated with human oncogenic herpesviruses. AP-1, activator protein-1; IL-1, interleukin-1; IL-1β, interleukin-1β; IL-6, interleukin-6; IL-8, interleukin-8; IL-10, interleukin-10; IL-12, interleukin-12; NF-κB, nuclear factor-kappa B; TNF-α, tumor necrosis factor-α.

The promising therapeutic application of BBR as an anti-inflammatory agent has come to light in recent years and is documented in many investigations [121,122]. NF-κB and activator protein-1 (AP-1) signaling pathways and pro-inflammatory cytokines, such as interleukins (1, 1β, 6, 8, 10, and 12) and tumor necrosis factor-α (TNF-α), were detected to play critical functions in inflammation allied with HOHV infections [35,109]. The main mechanisms of BBR were found to be related to the inhibition of the NF-κB and AP-1 pathways and suppression of the expression of pro-inflammatory cytokines (Figure 3).

## 6. Berberine Safety Profile

BBR is a herbal product that is generally safe and well tolerated. It is widely used as a dietary supplement and is marketed as a commercial product in powder or capsule form [123,124]. There are currently no proven dosages for BBR, especially for curing microbial infections, including herpesvirus diseases. However, most studies have recommended effective doses of 1–1.5 g/day for treating certain conditions such as diabetes type 2, hyperlipemia, and hypertension [125,126]. BBR has a half-life of several hours and hence does not remain in the body for a long time. Most marketed BBR supplements (commercial products) contain 0.5 g/capsule, and supplement labels frequently advise taking BBR three times per day before meals [127,128]. Preclinical experiments have confirmed the capacity of BBR to prevent undesirable toxic reactions generated by antitumor drugs such as cisplatin, cyclophosphamide, doxorubicin, and bleomycin [129,130,131,132]. Although the safety profile of BBR is high, there is a need for consultation with a healthcare provider before adding it to a daily routine.

On the other hand, some cautions have been reported with BBR use. For example, BBR may induce digestive adverse effects such as diarrhea, constipation, flatulence, and stomach pain [133]. Antimicrobial macrolides were found to interact with BBR, leading to potentially serious arrhythmias [134]. A cardiotoxic effect was detected when BBR co-administered with statins by a mechanism via suppressing CYP3A4 and human ether-a-go-go-related genes (hERG) potassium channels [135]. Additionally, BBR is not recommended during pregnancy and lactation [16]. The toxicity of pure BBR has been claimed in some studies to be greater than the toxicity of plant extracts/fractions containing BBR [136].

## 7. Strategies Involving Berberine for Improving Herpesvirus Therapy

HOHVs are known to use diverse strategies to control the host immune system and prevent it from initiating the required defense mechanisms. This powerful control enables the virus to regulate the genome of the host cell and subsequently generate latent and lytic infections [35]. Hence, several strategies/technologies have been designed to combat herpesvirus diseases. These approaches can also involve anti-herpesvirus natural drugs (such as BBR) to boost the therapeutic effectiveness.

### 7.1. Technologies to Improve BBR Bioavailability

BBR has been shown to help improve a range of conditions in humans; however, its low solubility and weak oral bioavailability significantly restrict its clinical use [137]. To overcome such barriers, this molecule has been produced in specific forms such as BBR chloride and BBR sulfate with improved oral bioavailability, allowing them to be used clinically [138]. Other attempts were also made to enhance BBR pharmacokinetics and hence its therapeutic efficacy using chemical modification procedures such as total or combinatorial synthesis [139]. During the past decade, huge efforts have been made to improve the drug delivery system of BBR with the hope to enhance its bioavailability and maximize the medicinal properties. Since this paper focuses on HOHVs and the associated cancers, in this section, we will emphasize advances that have been achieved in cancer treatment. Unfortunately, no studies have been performed on herpesviruses so far.

Numerous nano-strategies for BBR delivery were designed with promising tumor therapy. For instance, BBR-loaded lipid-based nanocarriers, such as solid lipid nanoparticles (SLNs), nanostructured lipid carriers (NLCs), and liposomes, showed improved antitumor action [140,141,142,143]. Moreover, BBR combined with various nanocarriers including dendrimer, graphene, Au, and silver nanoparticles demonstrated enhanced anticancer properties [144,145]. Recently, two studies have designed technologies to improve BBR delivery using hydrogel systems with promising pharmaceutical applications. pH-responsive composite hydrogel beads were described as a potential delivery carrier for the controlled release of BBR hydrochloride in the gastrointestinal tract [146]. Besides, the silk-sericin-derived hydrogel was demonstrated to be proper for a long-term release of BBR [147].

Since the reviewed strategies were performed at the level of preclinical experiments, further studies should be carried out to validate their efficacy in clinical trials. Moreover, extensive investigations on herpesviruses are greatly required.

### 7.2. Strategies Targeting the Physical Properties of Herpesvirus

The current method to treat herpesvirus infections targets viral proteins (such as herpesvirus DNA polymerase), thus inhibiting the viral replication and subsequently preventing recurrent infection. This means that all current treatment regimens target the molecular properties of the virus. It is known that the course of viral infection is determined by the molecular and physical properties of the virus. However, the physical properties have received little consideration so far [148,149].

Recently, Brandariz-Nuñez and his research team [150] have discovered a new and broad-spectrum approach to treat human herpesviruses, including the resistant strains. This approach targets physical properties in the genome of the virus and enables drugs to penetrate the protein shell of the virus and stop genes from leaving the virus to infect the cell. This method does not lead to resistance and works independently of mutations in the genome of the virus. Briefly, the research team found that herpesvirus has high internal pressure (20 atmospheres) because it is tightly packed with genetic material. This pressure permits the virus to infect a cell by ejecting its genes at a high speed into the cell nucleus after penetrating the cell. Therefore, their discovery is based on lowering or turning off the pressure in the genome of the virus without damaging the cell, allowing antiherpetic drugs to effectively fight against the virus [150].

The promising efficacy of this approach might enable drugs that show an acceptable level of anti-herpesvirus properties such as BBR to be involved in further investigations at the animal and human levels.

### 7.3. CRISPR/Cas9 Genome Editing Technique

Targeting the viral genetic components necessary for herpesvirus fitness by using CRISPR/Cas9 genome editing technology has made the aim to effectively manage and prevent herpesvirus infections achievable [151]. This technique has been shown to successfully block both productive and latent HOHVs (EBV, KSHV, HSV, and HCMV) infections, thereby stopping viral production from infected cells through inhibiting viral replication [152,153]. However, this technology needs to be involved in further studies for the verification of its efficacy in clinical practice against HOHVs. Treatment that consists of the CRISPR/Cas9 technique and pharmacotherapy (including natural anti-herpesvirus drugs such as BBR and others) may open new gates for the efficient eradication of HOHV infections. Therefore, accelerated efforts should be made to attain such a goal.

### 7.4. Combination Therapy for Herpesvirus Infections

Combination therapy has been proved to dramatically reduce the possibility of drug resistance and cytotoxicity. This has been observed in several infectious diseases, including herpesvirus infections [2,13]. Song and colleagues [100] revealed in vitro a significant synergy effect against HSV-2 when BBR combined with acyclovir with a combination index (CI = 0.814) was used. Acyclovir and other nucleoside analogs use a mechanism that targets viral DNA polymerase, which is essential for viral replication, while BBR targets diverse steps in the HOHV life cycle. This indicates that their use as a combinatory treatment with diverse mechanisms of action could lead to significant advances in the treatment of HOHV infections. On the other hand, some points should be considered while using such a strategy, particularly at the animal and human levels, such as non-toxic effective concentrations or doses and the possible adverse effects.

## 8. Conclusion and Take-Home Message

Oncogenic herpesvirus is challenging to treat because of fundamental problems related to latency, reactivation, and induction of cancer. The current treatment regimens can help cure the infection symptoms and suppress the viral replication but cannot entirely prevent the reactivation process or restrict the virus from establishing latency. Additionally, the long-term use of anti-herpesvirus drugs has caused the problem of drug resistance, leading to significant treatment failure. Novel anti-herpesvirus drugs with the ability to tackle such difficulties are urgently required.

In this review and based on a decent amount of in vitro and animal research, we highlighted the protective effects of BBR and how it may help manage HOHV infections and their associated cancers. We have also focused on presenting the mechanisms and pathways induced by BBR and its effective concentrations or doses responsible for the induced therapeutic effects. Furthermore, this review emphasized various strategies/technologies to increase BBR bioavailability along with other opportunities that could enhance its anti-herpesvirus properties. BBR, a multi-functional herbal medicine, showed in preclinical studies remarkable inhibition of multiple targets in the herpesvirus replication cycle. Since no clinical trials have been performed on BBR against HOHVs so far, there is a need to test this drug across various patient populations and conditions, including children and neonates, for whom limited treatment options currently exist. On the other hand, a few clinical trials incorporated BBR (BBR chloride form) to investigate its chemopreventive effect on developing colorectal cancer, a type of cancer associated with HOHV infections. These clinical trials were retrieved from (https://clinicaltrials.gov/ accessed on 21 May 2021) and are summarized in a supplementary table (Table S1; please refer to the supplementary file). The performed clinical studies are limited; therefore, further clinical investigations are needed to examine the preventive and therapeutic uses of BBR against HOHV-associated cancers.

Finally, since BBR is not an approved drug, we should remind the readers that BBR as a dietary supplement should be used with caution and under the supervision of a healthcare provider.

## Figures and Tables

**Table 1:** Protective effects of berberine against Epstein–Barr virus and its linked tumors.

Type of Study, Assay, Virus, and Cells/Animals	Outcomes	Mechanism of Action	Reference
In vitro. Viral titer and Western blotting assays. EBV. EBV-positive NPC cells (HONE1 and HK1-EBV cells).	At a concentration of 50 µM, BBR effectively reduced the production of virions in HONE1 and HK1-EBV cells, thus inhibiting latent and lytic replication of EBV in EBV-positive NPC cells.	BBR decreased the expression of the EBV transcription factor BZLF1.	[59]
In vitro and in vivo. Various biochemical assays. EBV-positive NPC cells (HONE1 and HK1-EBV cells). NOD/SCID mice.	At various concentrations in micromolar ranges, BBR successfully inhibited the viability of EBV-positive NPC cells and exposed cell cycle arrest and apoptosis in the EBV-positive NPC cells, providing a significant antitumor effect against NPC.	Reduction of EBNA1 expression and inhibition of STAT3 activation.	[59]
In vivo. Tumorigenicity, Western blot, and immunohistochemistry analyses. EBV-positive NPC cells (C666-1) in athymic nude mice.	Treatment with BBR at doses of 5 and 10 mg/kg significantly suppressed the tumorigenicity and growth of NPC cells.	Inhibition of STAT3 activation. Inhibition of IL-6-activated STAT3.	[60]
In vitro and in vivo. Cell proliferation, cell apoptosis, and Western blot assays. Xenograft tumor models of human NPC analysis. Male nude mice (BALB/C-NU).	Combined treatment of BBR (10 mg/kg) with Rg3 (5 mg/kg) remarkably diminished tumor growth in NPC CNE2 xenograft nude mice.	Enhancement of the expression of the apoptosis-associated protein Bax. Inhibition of survivin, PCNA, and anti-apoptotic protein Bcl-2 expressions via targeting the MAPK/ERK signaling pathways.	[63]
In vitro. Multiple biochemical assays. EBV-transformed B cells and cancerous B cells.	Treatment with BBR (50 µM) lessened cell viability and demonstrated apoptosis through a mitochondria-dependent pathway in EBV-transformed B cells and cancerous B cells.	The mechanism has been elucidated through p53-mediated regulation of XAF1 and GADD45α expressions.	[64]

The displayed mechanisms have been revealed by in vitro and in vivo studies. BBR, berberine; EBNA1, Epstein–Barr nuclear antigen 1; EBV, Epstein–Barr virus; GADD45α, growth arrest and DNA damage inducible alpha; IL-6, interleukin-6; MAPK/ERK, mitogen-activated protein kinase/extracellular signal-regulated kinase; NOD/SCID, non-obese diabetic/severe combined immunodeficient; NPC, nasopharyngeal carcinoma; PCNA, proliferating cell nuclear antigen; Rg3, ginsenoside Rg3; STAT3, signal transducer and activator of transcription 3; XAF1, X-linked inhibitor of apoptosis protein-associated factor 1.

**Table 2:** Antiviral effects of berberine and its derivatives against herpes simplex virus.

Type of Study, Assay, Virus, and Cells/Animals	Outcomes	Mechanism of Action	Reference
In vitro. Viral plaque assay coupled with multiple biochemical assays. HSV-1 and HSV-2. HEK_293_T, HEC-1-A, and Vero cells.	BBR blocked the replication of HSV-1 and HSV-2 with EC_50_s values of 6.77 and 5.04 µM, respectively.	Inhibition of IE gene expression. Reduction of HSV-induced NF-κB activation, as well as IκB-α degradation and p65 nuclear translocation. Inhibition of HSV-induced JNK phosphorylation.	[100]
In vitro. Plaque reduction, viral adsorption, viral penetration, cell viability (MTT), and Western blotting assays. HSV-1 and HSV-2. Vero cells.	HSV-1 and HSV-2 replications were impeded by BBR with IC_50_ values of 8.2 × 10^−2^ and 9.0 × 10^−2^ mg/mL, respectively. BBR inhibited HSV-1 and HSV-2 adsorption, with % inhibition of 93.2% and 93.9%, respectively. BBR had no significant inhibition impact on virus penetration.	The mechanism was assessed via inhibiting the late gene products gB and gE that play a fundamental role in HSV pathogenesis.	[101]
In vitro. Viral plaque and MTT assays. HSV-2. Human vaginal epithelial cells	BBR treatment (6.25 µM) showed slight inhibition of HSV-2 in human vaginal epithelial cells.	No mechanism of action was disclosed.	[102]
In vitro. Viral plaque, TBE, and immunoblot assays. HSV-GFP. Immune RAW264.7 cells.	BBR (10 µg/mL) inhibited GFP expression and reduced viral titers by 3-fold.	A mechanism that affects type I IFN stimulation was suggested.	[103]
In vitro. Cytopathic effect inhibition assay HSV-1 and HSV-2. Vero cells.	HB-13 lessened the activity of HSV-1 and HSV-2 with IC_50_ values of 1.33 and 1.34 µg/mL, respectively.	No mechanism of action was revealed.	[104]
In vitro and in vivo. Multiple analytical and bioanalytical techniques. Herpes lesions (only symptom of HSV infection). Pig model.	HB-13, in a gel formulation (0.5%), was investigated in a pig model and exhibited promising application in the treatment of herpes lesions. The effective concentration was found to be 2.51 µg/mL.	No mechanism of action was indicated.	[105]

BBR, berberine; EC_50_s, 50% maximal effective concentration; GFP, green fluorescent protein; gB, glycoprotein B; gE, glycoprotein E; HB-13, 13-hexyl-berberine hydrochloride; HSV-1, herpes simplex virus 1; HSV-2, herpes simplex virus 2; IFN, interferon; IC_50_, 50% inhibitory concentration; IE, immediate-early; JNK, c-Jun N-terminal kinase; MTT, 3-(4,5-dimethylthiazol-2-yl)-2,5-diphenyl tetrazolium bromide; NF-κB, nuclear factor kappa B; TBE, trypan blue exclusion. All reported mechanisms have been disclosed by in vitro experiments.

**Table 3:** Antiviral effect of berberine against human cytomegalovirus.

Type of Study, Assay, Virus, and Cells	Outcomes	Mechanism of Action	Reference
In vitro. Plaque reduction, MTT, qPCR, immunoblotting, cell transfection, and adenoviral transduction assays. Various HCMV strains (laboratory strain, clinical isolates, and drug-resistant strains). Human foreskin fibroblast (HFF) cells.	BBR potently inhibited the replication of all test strains with EC_50_ values ranging from 1.3 to 4.0 µM.	BBR interferes with the viral IE2 protein transactivating activity.	[106]
In vitro. Plaque reduction assay. HCMV. MRC-5 cells.	BBR chloride (an orally available form of BBR) efficiently inhibited the replication of HCMV with an IC_50_ value of 0.68 µM.	The mechanism was proposed: via interfering with intracellular functions after virus penetration into the host cells and before viral DNA synthesis.	[108]

BBR, berberine; EC_50_, 50% effective concentration; HCMV, human cytomegalovirus; IE2, immediate early-2.; IC_50_, 50% inhibitory concentration; MTT, 3-(4,5-dimethylthiazol-2-yl)-2,5-diphenyl tetrazolium bromide; qPCR, quantitative real-time PCR. The reported mechanisms have been emphasized by in vitro studies.

## Data Availability

Not applicable.

## Supplementary Materials

The following is available online at https://www.mdpi.com/article/10.3390/v13061014/s1: Table S1: Berberine in cancer clinical trials.

## References

[1] Hassan S.T., Masarčíková R., Berchová K. (2015). Bioactive natural products with anti-herpes simplex virus properties. J. Pharm. Pharmacol..

[2] Treml J., Gazdová M., Šmejkal K., Šudomová M., Kubatka P., Hassan S.T.S. (2020). Natural Products-Derived Chemicals: Breaking Barriers to Novel Anti-HSV Drug Development. Viruses.

[3] Manners O., Murphy J.C., Coleman A., Hughes D.J., Whitehouse A. (2018). Contribution of the KSHV and EBV lytic cycles to tumourigenesis. Curr. Opin. Virol..

[4] Dittmer D.P., Damania B., Sin S.H. (2015). Animal models of tumorigenic herpesviruses--An update. Curr. Opin. Virol..

[5] Wołącewicz M., Becht R., Grywalska E., Niedźwiedzka-Rystwej P. (2020). Herpesviruses in Head and Neck Cancers. Viruses.

[6] Tomkins A., White C., Higgins S.P. (2015). Primary herpes simplex virus infection mimicking cervical cancer. BMJ Case Rep..

[7] Herbein G. (2018). The Human Cytomegalovirus, from Oncomodulation to Oncogenesis. Viruses.

[8] Glaunsinger B.A. (2015). Modulation of the Translational Landscape During Herpesvirus Infection. Annu. Rev. Virol..

[9] Asha K., Sharma-Walia N. (2021). Targeting Host Cellular Factors as a Strategy of Therapeutic Intervention for Herpesvirus Infections. Front. Cell Infect. Microbiol..

[10] Poole C.L., James S.H. (2018). Antiviral Therapies for Herpesviruses: Current Agents and New Directions. Clin. Ther..

[11] Hassan S.T.S. (2020). Brassicasterol with Dual Anti-Infective Properties against HSV-1 and *Mycobacterium tuberculosis*, and Cardiovascular Protective Effect: Nonclinical In Vitro and In Silico Assessments. Biomedicines.

[12] Brezáni V., Leláková V., Hassan S.T.S., Berchová-Bímová K., Nový P., Klouček P., Maršík P., Dall’Acqua S., Hošek J., Šmejkal K. (2018). Anti-Infectivity against Herpes Simplex Virus and Selected Microbes and Anti-Inflammatory Activities of Compounds Isolated from *Eucalyptus globulus* Labill. Viruses.

[13] Hassan S.T.S., Šudomová M., Berchová-Bímová K., Šmejkal K., Echeverría J. (2019). Psoromic Acid, a Lichen-Derived Molecule, Inhibits the Replication of HSV-1 and HSV-2, and Inactivates HSV-1 DNA Polymerase: Shedding Light on Antiherpetic Properties. Molecules.

[14] Čulenová M., Sychrová A., Hassan S.T.S., Berchová-Bímová K., Svobodová P., Helclová A., Michnová H., Hošek J., Vasilev H., Suchý P. (2020). Multiple In vitro biological effects of phenolic compounds from *Morus alba* root bark. J. Ethnopharmacol..

[15] Hassan S.T.S., Švajdlenka E. (2017). Biological Evaluation and Molecular Docking of Protocatechuic Acid from *Hibiscus sabdariffa* L. as a Potent Urease Inhibitor by an ESI-MS Based Method. Molecules.

[16] Feng X., Sureda A., Jafari S., Memariani Z., Tewari D., Annunziata G., Barrea L., Hassan S.T.S., Šmejkal K., Malaník M. (2019). Berberine in Cardiovascular and Metabolic Diseases: From Mechanisms to Therapeutics. Theranostics.

[17] Wang K., Feng X., Chai L., Cao S., Qiu F. (2017). The metabolism of berberine and its contribution to the pharmacological effects. Drug Metab. Rev..

[18] Warowicka A., Nawrot R., Goździcka-Józefiak A. (2020). Antiviral activity of berberine. Arch. Virol..

[19] Zeng Q., Deng H., Li Y., Fan T., Liu Y., Tang S., Wei W., Liu X., Guo X., Jiang J. (2021). Berberine Directly Targets the NEK7 Protein to Block the NEK7-NLRP3 Interaction and Exert Anti-inflammatory Activity. J. Med. Chem..

[20] Liu D., Meng X., Wu D., Qiu Z., Luo H. (2019). A Natural Isoquinoline Alkaloid with Antitumor Activity: Studies of the Biological Activities of Berberine. Front. Pharmacol..

[21] Hassan S.T.S. (2020). Shedding Light on the Effect of Natural Anti-Herpesvirus Alkaloids on SARS-CoV-2: A Treatment Option for COVID-19. Viruses.

[22] Johnston B.P., McCormick C. (2019). Herpesviruses and the Unfolded Protein Response. Viruses.

[23] Stempel M., Chan B., Brinkmann M.M. (2019). Coevolution pays off: Herpesviruses have the license to escape the DNA sensing pathway. Med. Microbiol. Immunol..

[24] Adler B., Sattler C., Adler H. (2017). Herpesviruses and Their Host Cells: A Successful Liaison. Trends Microbiol..

[25] Jarosinski K.W. (2017). Interindividual Spread of Herpesviruses. Adv. Anat. Embryol. Cell Biol..

[26] Azab W., Dayaram A., Greenwood A.D., Osterrieder N. (2018). How Host Specific Are Herpesviruses? Lessons from Herpesviruses Infecting Wild and Endangered Mammals. Annu. Rev. Virol..

[27] Lomonte P. (2017). Herpesvirus Latency: On the Importance of Positioning Oneself. Adv. Anat. Embryol. Cell Biol..

[28] Cohen J.I. (2020). Herpesvirus latency. J. Clin. Investig..

[29] Connolly S.A., Jardetzky T.S., Longnecker R. (2021). The structural basis of herpesvirus entry. Nat. Rev. Microbiol..

[30] Sadeghipour S., Mathias R.A. (2017). Herpesviruses hijack host exosomes for viral pathogenesis. Semin. Cell. Dev. Biol..

[31] Choi U.Y., Park A., Jung J.U. (2017). Double the Trouble When Herpesviruses Join Hands. Cell Host Microbe.

[32] Ho D.Y., Enriquez K., Multani A. (2020). Herpesvirus Infections Potentiated by Biologics. Infect. Dis. Clin. N. Am..

[33] Koyuncu O.O., MacGibeny M.A., Enquist L.W. (2018). Latent versus productive infection: The alpha herpesvirus switch. Future Virol..

[34] Lagunoff M. (2016). Activation of cellular metabolism during latent Kaposi’s Sarcoma herpesvirus infection. Curr. Opin. Virol..

[35] Šudomová M., Hassan S.T.S. (2021). Nutraceutical Curcumin with Promising Protection against Herpesvirus Infections and Their Associated Inflammation: Mechanisms and Pathways. Microorganisms.

[36] Thorley-Lawson D.A. (2015). EBV Persistence--Introducing the Virus. Curr. Top. Microbiol. Immunol..

[37] Zaman A., Rahaman M.H., Razzaque S. (2013). Kaposi’s sarcoma: A computational approach through protein-protein interaction and gene regulatory networks analysis. Virus Genes.

[38] Li R., Liao G., Nirujogi R.S., Pinto S.M., Shaw P.G., Huang T.C., Wan J., Qian J., Gowda H., Wu X. (2015). Phosphoproteomic Profiling Reveals Epstein-Barr Virus Protein Kinase Integration of DNA Damage Response and Mitotic Signaling. PLoS Pathog..

[39] Baquero-Pérez B., Whitehouse A. (2015). Hsp70 Isoforms Are Essential for the Formation of Kaposi’s Sarcoma-Associated Herpesvirus Replication and Transcription Compartments. PLoS Pathog..

[40] Li D.J., Verma D., Mosbruger T., Swaminathan S. (2014). CTCF and Rad21 act as host cell restriction factors for Kaposi’s sarcoma-associated herpesvirus (KSHV) lytic replication by modulating viral gene transcription. PLoS Pathog..

[41] Li Q., Wilkie A.R., Weller M., Liu X., Cohen J.I. (2015). THY-1 Cell Surface Antigen (CD90) Has an Important Role in the Initial Stage of Human Cytomegalovirus Infection. PLoS Pathog..

[42] Weekes M.P., Tomasec P., Huttlin E.L., Fielding C.A., Nusinow D., Stanton R.J., Wang E.C.Y., Aicheler R., Murrell I., Wilkinson G.W.G. (2014). Quantitative temporal viromics: An approach to investigate host-pathogen interaction. Cell.

[43] Griffiths S.J., Koegl M., Boutell C., Zenner H.L., Crump C.M., Pica F., Gonzalez O., Friedel C.C., Barry G., Martin K. (2013). A systematic analysis of host factors reveals a Med23-interferon-λ regulatory axis against herpes simplex virus type 1 replication. PLoS Pathog..

[44] Griffiths S.J. (2013). Screening for host proteins with pro- and antiviral activity using high-throughput RNAi. Methods Mol. Biol..

[45] Münz C. (2019). Latency and lytic replication in Epstein-Barr virus-associated oncogenesis. Nat. Rev. Microbiol..

[46] Charostad J., Nakhaie M., Dehghani A., Faghihloo E. (2020). The interplay between EBV and KSHV viral products and NF-κB pathway in oncogenesis. Infect. Agents Cancer..

[47] Young L.S., Yap L.F., Murray P.G. (2016). Epstein-Barr virus: More than 50 years old and still providing surprises. Nat. Rev. Cancer.

[48] Epstein M.A., Achong B.G., Barr Y.M. (1964). Virus particles in cultured lymphoblasts from Burkitt’s lymphoma. Lancet.

[49] Epstein M.A., Henle G., Achong B.G., Barr Y.M. (1964). Morphological and biological studies on a virus in cultured lymphoblasts from Burkitt’s lymphoma. J. Exp. Med..

[50] Farrell P.J. (2019). Epstein–Barr virus and cancer. Annu. Rev. Pathol..

[51] Ciccarese G., Trave I., Herzum A., Parodi A., Drago F. (2020). Dermatological manifestations of Epstein-Barr virus systemic infection: A case report and literature review. Int. J. Dermatol..

[52] Cui Q., Feng F.T., Xu M., Liu W.S., Yao Y.Y., Xie S.H., Li X.Z., Ye Z.L., Feng Q.S., Chen L.Z. (2016). Nasopharyngeal carcinoma risk prediction via salivary detection of host and Epstein-Barr virus genetic variants. Oncotarget.

[53] Xu M., Cheung C.C., Chow C., Lun S.W., Cheung S.T., Lo K.W. (2016). Overexpression of PIN1 enhances cancer growth and aggressiveness with cyclin D1 induction in EBV-associated nasopharyngeal carcinoma. PLoS ONE..

[54] Wang F.W., Wu X.R., Liu W.J., Liang Y.J., Huang Y.F., Liao Y.J., Shao C.K., Zong Y.S., Mai S.J., Xie D. (2012). The nucleotide polymorphisms within the Epstein-Barr virus C and Q promoters from nasopharyngeal carcinoma affect transcriptional activity in vitro. Eur. Arch. Otorhinolaryngol..

[55] Shen Y., Zhang S., Sun R., Wu T., Qian J. (2015). Understanding the interplay between host immunity and Epstein-Barr virus in NPC patients. Emerg. Microbes Infect..

[56] Kelly G.L., Stylianou J., Rasaiyaah J., Wei W., Thomas W., Croom-Carter D., Kohler C., Spang R., Woodman C., Kellam P. (2013). Different patterns of Epstein-Barr virus latency in endemic Burkitt lymphoma (BL) lead to distinct variants within the BL-associated gene expression signature. J. Virol..

[57] Kempkes B., Ling P.D. (2015). EBNA2 and Its Coactivator EBNA-LP. Curr. Top. Microbiol. Immunol..

[58] Frappier L. (2012). Contributions of Epstein-Barr nuclear antigen 1 (EBNA1) to cell immortalization and survival. Viruses.

[59] Wang C., Wang H., Zhang Y., Guo W., Long C., Wang J., Liu L., Sun X. (2017). Berberine inhibits the proliferation of human nasopharyngeal carcinoma cells via an Epstein-Barr virus nuclear antigen 1-dependent mechanism. Oncol. Rep..

[60] Tsang C.M., Cheung Y.C., Lui V.W., Yip Y.L., Zhang G., Lin V.W., Cheung K.C., Feng Y., Tsao S.W. (2013). Berberine suppresses tumorigenicity and growth of nasopharyngeal carcinoma cells by inhibiting STAT3 activation induced by tumor associated fibroblasts. BMC Cancer.

[61] Tao D., Zhang N., Huang Q., Ge C., Li Q., Li S., Weng K., Guo Q., Sui J., Wang C. (2020). Association of Epstein-Barr virus infection with peripheral immune parameters and clinical outcome in advanced nasopharyngeal carcinoma. Sci. Rep..

[62] Hassan S.T.S., Berchová-Bímová K., Petráš J., Hassan K.T.S. (2017). Cucurbitacin B interacts synergistically with antibiotics against *Staphylococcus aureus* clinical isolates and exhibits antiviral activity against HSV-1. S. Afr. J. Bot..

[63] Zhou F., Hu J., Dai N., Song L., Lin T., Liu J., Li K., Peng Z., He Y., Liao D.-F. (2020). Berberine and ginsenoside Rg3 act synergistically via the MAPK/ERK pathway in nasopharyngeal carcinoma cells. J. Funct. Foods.

[64] Park G.B., Park S.H., Kim D., Kim Y.S., Yoon S.H., Hur D.Y. (2016). Berberine induces mitochondrial apoptosis of EBV-transformed B cells through p53-mediated regulation of XAF1 and GADD45α. Int. J. Oncol..

[65] Kumar B., Roy A., Veettil M.V., Chandran B. (2018). Insight into the Roles of E3 Ubiquitin Ligase c-Cbl, ESCRT Machinery, and Host Cell Signaling in Kaposi’s Sarcoma-Associated Herpesvirus Entry and Trafficking. J. Virol..

[66] Minhas V., Wood C. (2014). Epidemiology and transmission of Kaposi’s sarcoma-associated herpesvirus. Viruses.

[67] Ueda K. (2018). KSHV Genome Replication and Maintenance in Latency. Adv. Exp. Med. Biol..

[68] Li S., Bai L., Dong J., Sun R., Lan K. (2017). Kaposi’s Sarcoma-Associated Herpesvirus: Epidemiology and Molecular Biology. Adv. Exp. Med. Biol..

[69] Schneider J.W., Dittmer D.P. (2017). Diagnosis and Treatment of Kaposi Sarcoma. Am. J. Clin. Dermatol..

[70] Watanabe T., Sugimoto A., Hosokawa K., Fujimuro M. (2018). Signal Transduction Pathways Associated with KSHV-Related Tumors. Adv. Exp. Med. Biol..

[71] Abere B., Mamo T.M., Hartmann S., Samarina N., Hage E., Rückert J., Hotop S.K., Büsche G., Schulz T.F. (2017). The Kaposi’s sarcoma-associated herpesvirus (KSHV) non-structural membrane protein K15 is required for viral lytic replication and may represent a therapeutic target. PLoS Pathog..

[72] Cesarman E., Damania B., Krown S.E., Martin J., Bower M., Whitby D. (2019). Kaposi sarcoma. Nat. Rev. Dis. Primers.

[73] Shimada K., Hayakawa F., Kiyoi H. (2018). Biology and management of primary effusion lymphoma. Blood.

[74] Goto H., Kariya R., Shimamoto M., Kudo E., Taura M., Katano H., Okada S. (2012). Antitumor effect of berberine against primary effusion lymphoma via inhibition of NF-κB pathway. Cancer Sci..

[75] Damania B., Münz C. (2019). Immunodeficiencies that predispose to pathologies by human oncogenic γ-herpesViruses. FEMS Microbiol. Rev..

[76] Tada S., Hamada M., Yura Y. (2018). Proteomic Analysis of Secretomes of Oncolytic Herpes Simplex Virus-Infected Squamous Cell Carcinoma Cells. Cancers.

[77] Liljeqvist J.Å., Tunbäck P., Norberg P. (2009). Asymptomatically shed recombinant herpes simplex virus type 1 strains detected in saliva. J. Gen. Virol..

[78] Kameyama T., Haikata K., Nakamura Y., Murase H., Yamamoto S. (1989). Shedding of herpes simplex virus type 1 into saliva after surgery for oral and genital or urological cancer patients. Kurume Med. J..

[79] Nolan A. (2009). Interventions for prevention and treatment of herpes simplex virus in cancer patients. Evid. Based Dent..

[80] Correia A.V., Coêlho M.R., de Oliveira Mendes Cahú G.G., de Almeida Silva J.L., da Mota Vasconcelos Brasil C., de Castro J.F. (2015). Seroprevalence of HSV-1/2 and correlation with aggravation of oral mucositis in patients with squamous cell carcinoma of the head and neck region submitted to antineoplastic treatment. Support Care Cancer.

[81] Smith J.W., Torres J.E., Holmquist N.D. (1979). Association of Herpes simplex virus (HSV) with cervical cancer by lymphocyte reactivity with HSV-1 and HSV-2 antigens. Am. J. Epidemiol..

[82] Thomas F., Elguero E., Brodeur J., Le Goff J., Missé D. (2011). Herpes simplex virus type 2 and cancer: A medical geography approach. Infect. Genet. Evol..

[83] Parker T.M., Smith E.M., Ritchie J.M., Haugen T.H., Vonka V., Turek L.P., Hamsikova E. (2006). Head and neck cancer associated with herpes simplex virus 1 and 2 and other risk factors. Oral Oncol..

[84] Schildt E.B., Eriksson M., Hardell L., Magnuson A. (1998). Oral infections and dental factors in relation to oral cancer: A Swedish case--control study. Eur. J. Cancer Prev..

[85] Starr J.R., Daling J.R., Fitzgibbons E.D., Madeleine M.M., Ashley R., Galloway D.A., Schwartz S.M. (2001). Serologic evidence of herpes simplex virus 1 infection and oropharyngeal cancer risk. Cancer Res..

[86] Michaelis M., Doerr H.W., Cinatl J. (2009). The story of human cytomegalovirus and cancer: Increasing evidence and open questions. Neoplasia.

[87] Ahmed H.G., Suliman R.S.A., Ashankyty I.M., Albieh Z.A., Warille A.A. (2018). Role of human Cytomegalovirus in the etiology of nasopharyngeal carcinoma. J. Cancer Res. Ther..

[88] Kiprian D., Czarkowska-Paczek B., Wyczalkowska-Tomasik A., Paczek L. (2018). Human cytomegalovirus and Epstein-Barr virus infections increase the risk of death in patients with head and neck cancers receiving radiotherapy or radiochemotherapy. Medicine.

[89] Richardson A.K., Walker L.C., Cox B., Rollag H., Robinson B.A., Morrin H., Pearson J.F., Potter J.D., Paterson M., Surcel H.M. (2020). Breast cancer and cytomegalovirus. Clin. Transl. Oncol..

[90] Zhang L., Guo G., Xu J., Sun X., Chen W., Jin J., Hu C., Zhang P., Shen X., Xue X. (2017). Human cytomegalovirus detection in gastric cancer and its possible association with lymphatic metastasis. Diagn. Microbiol. Infect. Dis..

[91] Lawler S.E. (2015). Cytomegalovirus and glioblastoma; controversies and opportunities. J. Neurooncol..

[92] Teo W.H., Chen H.P., Huang J.C., Chan Y.J. (2017). Human cytomegalovirus infection enhances cell proliferation, migration and upregulation of EMT markers in colorectal cancer-derived stem cell-like cells. Int. J. Oncol..

[93] Golais F., Mrázová V. (2020). Human alpha and beta herpesviruses and cancer: Passengers or foes?. Folia Microbiol..

[94] Dziurzynski K., Chang S.M., Heimberger A.B., Kalejta R.F., McGregor Dallas S.R., Smit M., Soroceanu L., Cobbs C.S. (2012). HCMV and Gliomas Symposium. Consensus on the role of human cytomegalovirus in glioblastoma. Neuro Oncol..

[95] Blaylock R.L. (2019). Accelerated cancer aggressiveness by viral oncomodulation: New targets and newer natural treatments for cancer control and treatment. Surg. Neurol. Int..

[96] Chen H.P., Chan Y.J. (2014). The oncomodulatory role of human cytomegalovirus in colorectal cancer: Implications for clinical trials. Front. Oncol..

[97] Hassan S.T.S., Šudomová M., Masarčíková R. (2017). Herpes simplex virus infection: An overview of the problem, pharmacologic therapy and dietary measures. Ceska Slov. Farm..

[98] Zhao J., Qin C., Liu Y., Rao Y., Feng P. (2021). Herpes Simplex Virus and Pattern Recognition Receptors: An Arms Race. Front. Immunol..

[99] Sawtell N.M., Thompson R.L. (2021). Alphaherpesvirus Latency and Reactivation with a Focus on Herpes Simplex Virus. Curr. Issues Mol. Biol..

[100] Song S., Qiu M., Chu Y., Chen D., Wang X., Su A., Wu Z. (2014). Downregulation of cellular c-Jun N-terminal protein kinase and NF-κB activation by berberine may result in inhibition of herpes simplex virus replication. Antimicrob. Agents Chemother..

[101] Chin L.W., Cheng Y.W., Lin S.S., Lai Y.Y., Lin L.Y., Chou M.Y., Chou M.C., Yang C.C. (2010). Anti-herpes simplex virus effects of berberine from *Coptidis rhizoma*, a major component of a Chinese herbal medicine, Ching-Wei-San. Arch. Virol..

[102] Duan Q., Liu T., Yuan P., Huang C., Shao Q., Xu L., Sun J., Huang G., Chen Z. (2020). Antiviral effect of Chinese herbal prescription JieZe-1 on adhesion and penetration of VK2/E6E7 with herpes simplex viruses type 2. J. Ethnopharmacol..

[103] Kim J.H., Weeratunga P., Kim M.S., Nikapitiya C., Lee B.H., Uddin M.B., Kim T.H., Yoon J.E., Park C., Ma J.Y. (2016). Inhibitory effects of an aqueous extract from *Cortex Phellodendri* on the growth and replication of broad-spectrum of viruses in vitro and in vivo. BMC Complement. Altern. Med..

[104] Wu J.B., Zheng J.R., Lin Z., Li X.Y., Cui P.G. (2007). In vitro antiviral activity of a berberine derivant HB-13 against herpes simplex virus. Chin. J. Dermatol..

[105] Wei H.L., Wang S., Xu F., Xu L.F., Zheng J.R., Chen Y. (2013). Evaluation of a 13-hexyl-berberine hydrochloride topical gel formulation. Drug Dev. Ind. Pharm..

[106] Luganini A., Mercorelli B., Messa L., Palù G., Gribaudo G., Loregian A. (2019). The isoquinoline alkaloid berberine inhibits human cytomegalovirus replication by interfering with the viral Immediate Early-2 (IE2) protein transactivating activity. Antiviral Res..

[107] Pignoloni B., Fionda C., Dell’Oste V., Luganini A., Cippitelli M., Zingoni A., Landolfo S., Gribaudo G., Santoni A., Cerboni C. (2016). Distinct Roles for Human Cytomegalovirus Immediate Early Proteins IE1 and IE2 in the Transcriptional Regulation of MICA and PVR/CD155 Expression. J. Immunol..

[108] Hayashi K., Minoda K., Nagaoka Y., Hayashi T., Uesato S. (2007). Antiviral activity of berberine and related compounds against human cytomegalovirus. Bioorg. Med. Chem. Lett..

[109] Bennett J.M., Glaser R., Malarkey W.B., Beversdorf D.Q., Peng J., Kiecolt-Glaser J.K. (2012). Inflammation and reactivation of latent herpesviruses in older adults. Brain Behav. Immun..

[110] Cruz-Muñoz M.E., Fuentes-Pananá E.M. (2018). Beta and Gamma Human Herpesviruses: Agonistic and Antagonistic Interactions with the Host Immune System. Front Microbiol..

[111] Lobo A.M., Agelidis A.M., Shukla D. (2019). Pathogenesis of herpes simplex keratitis: The host cell response and ocular surface sequelae to infection and inflammation. Ocul. Surf..

[112] Islam S.M.S., Sohn S. (2018). HSV-Induced Systemic Inflammation as an Animal Model for Behçet’s Disease and Therapeutic Applications. Viruses.

[113] Johnston C., Corey L. (2016). Current Concepts for Genital Herpes Simplex Virus Infection: Diagnostics and Pathogenesis of Genital Tract Shedding. Clin. Microbiol. Rev..

[114] Alomari N., Totonchy J. (2020). Cytokine-Targeted Therapeutics for KSHV-Associated Disease. Viruses.

[115] Polizzotto M.N., Uldrick T.S., Wyvill K.M., Aleman K., Marshall V., Wang V., Whitby D., Pittaluga S., Jaffe E.S., Millo C. (2016). Clinical Features and Outcomes of Patients with Symptomatic Kaposi Sarcoma Herpesvirus (KSHV)-associated Inflammation: Prospective Characterization of KSHV Inflammatory Cytokine Syndrome (KICS). Clin. Infect. Dis..

[116] Shrivastava G., León-Juárez M., García-Cordero J., Meza-Sánchez D.E., Cedillo-Barrón L. (2016). Inflammasomes and its importance in viral infections. Immunol. Res..

[117] Liu T., Zhang L., Joo D., Sun S.C. (2017). NF-κB signaling in inflammation. Signal Transduct. Target. Ther..

[118] Carty M., Guy C., Bowie A.G. (2020). Detection of viral infections by innate immunity. Biochem. Pharmacol..

[119] Crusz S.M., Balkwill F.R. (2015). Inflammation and cancer: Advance and new agents. Nat. Rev. Clin. Oncol..

[120] Murata M. (2018). Inflammation and cancer. Environ. Health Prev. Med..

[121] Zou K., Li Z., Zhang Y., Zhang H.Y., Li B., Zhu W.L., Shi J.Y., Jia Q., Li Y.M. (2017). Advances in the study of berberine and its derivatives: A focus on anti-inflammatory and anti-tumor effects in the digestive system. Acta Pharmacol. Sin..

[122] Ehteshamfar S.M., Akhbari M., Afshari J.T., Seyedi M., Nikfar B., Shapouri-Moghaddam A., Ghanbarzadeh E., Momtazi-Borojeni A.A. (2020). Anti-inflammatory and immune-modulatory impacts of berberine on activation of autoreactive T cells in autoimmune inflammation. J. Cell. Mol. Med..

[123] Di Pierro F., Bertuccioli A., Giuberti R., Saponara M., Ivaldi L. (2020). Role of a berberine-based nutritional supplement in reducing diarrhea in subjects with functional gastrointestinal disorders. Minerva Gastroenterol. Dietol..

[124] Funk R.S., Singh R.K., Winefield R.D., Kandel S.E., Ruisinger J.F., Moriarty P.M., Backes J.M. (2018). Variability in Potency among Commercial Preparations of Berberine. J. Diet. Suppl..

[125] Lan J., Zhao Y., Dong F., Yan Z., Zheng W., Fan J., Sun G. (2015). Meta-analysis of the effect and safety of berberine in the treatment of type 2 diabetes mellitus, hyperlipemia and hypertension. J. Ethnopharmacol..

[126] Dong H., Wang N., Zhao L., Lu F. (2012). Berberine in the treatment of type 2 diabetes mellitus: A systemic review and meta-analysis. Evid. Based Complement. Alternat. Med..

[127] Gupta P.K., Gurley B.J., Barone G., Hendrickson H.P. (2010). Clinical Pharmacokinetics and Metabolism of Berberine and Hydrastine Following an Oral Dose of Goldenseal Supplement. Planta Med..

[128] Gupta P.K., Hubbard M., Gurley B., Hendrickson H.P. (2009). Validation of a liquid chromatography-tandem mass spectrometric assay for the quantitative determination of hydrastine and berberine in human serum. J. Pharm. Biomed. Anal..

[129] Domitrović R., Cvijanović O., Pernjak-Pugel E., Skoda M., Mikelić L., Crnčević-Orlić Z. (2013). Berberine exerts nephroprotective effect against cisplatin-induced kidney damage through inhibition of oxidative/nitrosative stress, inflammation, autophagy and apoptosis. Food Chem. Toxicol..

[130] Germoush M.O., Mahmoud A.M. (2014). Berberine mitigates cyclophosphamide-induced hepatotoxicity by modulating antioxidant status and inflammatory cytokines. J. Cancer Res. Clin. Oncol..

[131] Hao G., Yu Y., Gu B., Xing Y., Xue M. (2015). Protective effects of berberine against doxorubicin-induced cardiotoxicity in rats by inhibiting metabolism of doxorubicin. Xenobiotica.

[132] Chitra P., Saiprasad G., Manikandan R., Sudhandiran G. (2013). Berberine attenuates bleomycin induced pulmonary toxicity and fibrosis via suppressing NF-κB dependant TGF-β activation: A biphasic experimental study. Toxicol. Lett..

[133] Yin J., Xing H., Ye J. (2008). Efficacy of berberine in patients with type 2 diabetes mellitus. Metabolism.

[134] Zhi D., Feng P.F., Sun J.L., Guo F., Zhang R., Zhao X., Li B.X. (2015). The enhancement of cardiac toxicity by concomitant administration of Berberine and macrolides. Eur. J. Pharm. Sci..

[135] Feng P., Zhao L., Guo F., Zhang B., Fang L., Zhan G., Xu X., Fang Q., Liang Z., Li B. (2018). The enhancement of cardiotoxicity that results from inhibition of CYP 3A4 activity and hERG channel by berberine in combination with statins. Chem. Biol. Interact..

[136] Singh N., Sharma B. (2018). Toxicological Effects of Berberine and Sanguinarine. Front. Mol. Biosci..

[137] Habtemariam S. (2020). Berberine pharmacology and the gut microbiota: A hidden therapeutic link. Pharmacol Res..

[138] Hou Q., He W.J., Wu Y.S., Hao H.J., Xie X.Y., Fu X.B. (2020). Berberine: A Traditional Natural Product with Novel Biological Activities. Altern. Ther. Health Med..

[139] Gaba S., Saini A., Singh G., Monga V. (2021). An insight into the medicinal attributes of berberine derivatives: A review. Bioorg. Med. Chem..

[140] Wang L., Li H., Wang S., Liu R., Wu Z., Wang C., Wang Y., Chen M. (2014). Enhancing the antitumor activity of berberine hydrochloride by solid lipid nanoparticle encapsulation. AAPS PharmSciTech.

[141] Wang Z.P., Wu J.B., Chen T.S., Zhou Q., Wang Y.F. (2015). In vitro and in vivo antitumor efficacy of berberine-nanostructured lipid carriers against H22 tumor. Biophotonics and Immune Responses X.

[142] Lin Y.C., Kuo J.Y., Hsu C.C., Tsai W.C., Li W.C., Yu M.C., Wen H.W. (2013). Optimizing manufacture of liposomal berberine with evaluation of its antihepatoma effects in a murine xenograft model. Int. J. Pharm..

[143] Nguyen T.X., Huang L., Liu L., Elamin Abdalla A.M., Gauthier M., Yang G. (2014). Chitosan-coated nano-liposomes for the oral delivery of berberine hydrochloride. J. Mater. Chem. B.

[144] Mirhadi E., Rezaee M., Malaekeh-Nikouei B. (2018). Nano strategies for berberine delivery, a natural alkaloid of Berberis. Biomed. Pharmacother..

[145] Majidzadeh H., Araj-Khodaei M., Ghaffari M., Torbati M., Ezzati Nazhad Dolatabadi J., Hamblin M.R. (2020). Nano-based delivery systems for berberine: A modern anti-cancer herbal medicine. Colloids Surf. B Biointerfaces.

[146] Gao J., Fan D., Song P., Zhang S., Liu X. (2020). Preparation and application of pH-responsive composite hydrogel beads as potential delivery carrier candidates for controlled release of berberine hydrochloride. R. Soc. Open Sci..

[147] Yan C., Liang J., Fang H., Meng X., Chen J., Zhong Z., Liu Q., Hu H., Zhang X. (2021). Fabrication and Evaluation of Silk Sericin-Derived Hydrogel for the Release of the Model Drug Berberine. Gels.

[148] Brandariz-Nuñez A., Liu T., Du T., Evilevitch A. (2019). Pressure-driven release of viral genome into a host nucleus is a mechanism leading to herpes infection. Elife.

[149] Bauer D.W., Li D., Huffman J., Homa F.L., Wilson K., Leavitt J.C., Casjens S.R., Baines J., Evilevitch A. (2015). Exploring the Balance between DNA Pressure and Capsid Stability in Herpesviruses and Phages. J. Virol..

[150] Brandariz-Nuñez A., Robinson S.J., Evilevitch A. (2020). Pressurized DNA state inside herpes capsids-A novel antiviral target. PLoS Pathog..

[151] Van Diemen F.R., Kruse E.M., Hooykaas M.J., Bruggeling C.E., Schürch A.C., van Ham P.M., Imhof S.M., Nijhuis M., Wiertz E.J., Lebbink R.J. (2016). CRISPR/Cas9-Mediated Genome Editing of Herpesviruses Limits Productive and Latent Infections. PLoS Pathog..

[152] Van Diemen F.R., Lebbink R.J. (2017). CRISPR/Cas9, a powerful tool to target human herpesViruses. Cell Microbiol..

[153] Chen Y.C., Sheng J., Trang P., Liu F. (2018). Potential Application of the CRISPR/Cas9 System against Herpesvirus Infections. Viruses.

