# Supplementary material for: Berberine in Human Oncogenic Herpesvirus Infections and Their Linked Cancers
Source: Viruses. 2021 May 28;13(6):1014. doi: 10.3390/v13061014 (PMC8229678; doi:10.3390/v13061014)
Supplement: Supplementary file 1 [file viruses-13-01014-s001.zip › viruses-1206838-supplementary.pdf]

# ClinicalTrials.gov Search Results 05/21/2021

| | Title | Status | Study Results | Conditions | Interventions | Characteristics | Population | Dates | Locations |
|---|---|---|---|---|---|---|---|---|---|
| 1 | A Research of Berberine Hydrochloride to Prevent Colorectal Adenomas in Patients With Previous Colorectal Cancer | Unknown status | No Results Available | •Colorectal Adenomas | •Drug: Berberine hydrochloride<br>•Drug: Placebo | Phase:<br>•Phase 2<br>•Phase 3 | Enrollment: 1000<br>Age: 18 Years to 80 Years (Adult, Older Adult)<br>Sex: All | Study Completion: March 1, 2021 | •Xijing Hospital of Digestive Disease, Xi'an, Shaanxi, China |
| 2 | Study of Berberine Hydrochloride in Prevention of Colorectal Adenomas Recurrence | Completed | No Results Available | •Colorectal Adenoma | •Drug: Berberine hydrochloride<br>•Drug: placebo | Phase:<br>•Phase 2<br>•Phase 3 | Enrollment: 1108<br>Age: 18 Years to 75 Years (Adult, Older Adult)<br>Sex: All | Study Completion: December 29, 2018 | •Department of Gastroenterology, the Seventh Medical Center of PLA General Hospital, Beijing, Beijing, China<br>•Department of Gastroenterology, Zhongshan Hospital, Xiamen University, Xiamen, Fujian, China<br>•Department of Gastroenterology, Nanfang Hospital, Southern Medical Univerisity, Guangdong, Guangzhou, China<br>•Division of Gastroenterology and Hepatology, The Affiliated DrumTower of Nanjing University Medical School, Nanjing, Jiangsu, China<br>•Division of Gastroenterology and Hepatology, Ren-Ji Hospital, Shanghai Jiao-Tong University School of Medicine, Shanghai Institute of Digestive Disease; Key Laboratory of Gastroenterology & Hepatology, Ministry of Health, Shanghai, Shanghai, China<br>•Department of Gastroenterology, the Shanghai Tenth People's Hospital, Tongji University, Shanghai, Shanghai, China<br>•Department of Gastroenterology and Hepatology, General Hospital, Tianjin Medical University, Tianjin, Tianjin, China |
| 3 | Primary Chemoprevention of Familial Adenomatous Polyposis With Berberine Hydrochloride | Recruiting | No Results Available | •Colorectal Adenomas | •Drug: 100mg Berberine hydrochloride<br>•Drug: 300mg Berberine hydrochloride<br>•Drug: Placebo Oral Tablet | Phase:<br>•Phase 2<br>•Phase 3 | Enrollment: 100<br>Age: 18 Years to 65 Years (Adult, Older Adult)<br>Sex: All | Study Completion: December 31, 2020 | •Xijing Hospital of Digestive Disease, Xi'an, Shaanxi, China |

| | Title | Status | Study Results | Conditions | Interventions | Characteristics | Population | Dates | Locations |
|---|---|---|---|---|---|---|---|---|---|
| 4 | Berberine Chloride in Preventing Colorectal Cancer in Patients With Ulcerative Colitis in Remission | Active, not recruiting | No Results Available | •Ulcerative Colitis | •Drug: Berberine Chloride<br>•Other: Laboratory Biomarker Analysis<br>•Other: Placebo Administration | Phase:<br>Phase 1 | Enrollment:<br>18<br>Age:<br>18 Years to 70 Years (Adult, Older Adult)<br>Sex:<br>All | | •Northwestern University, Chicago, Illinois, United States<br>•Fourth Military Medical University, Xi'an, Shaanxi, China |

U.S. National Library of Medicine | U.S. National Institutes of Health | U.S. Department of Health & Human Services
